# Editorial: Hallmark of cancer: replicative immortality

**DOI:** 10.3389/fonc.2023.1204094

**Published:** 2023-04-25

**Authors:** Susan M. Bailey

**Affiliations:** Department of Environmental & Radiological Health Sciences, Colorado State University, Fort Collins, CO, United States

**Keywords:** cancer, replicative immortality, telomeres, telomerase, immune response, senescence

It’s now been over two decades since the Hallmarks of Cancer proposed by Douglas Hanahan and Robert Weinberg were first published (1), during which time the landscape of cancer research has changed considerably. In addition to the original six biological capabilities put forward as being acquired during the multistep development of human tumors, which included enabling replicative immortality, the “next generation” of hallmarks introduced two emerging hallmarks and two enabling characteristics (2). The “new dimensions” of the Hallmarks of Cancer proposed most recently incorporates additional emerging hallmarks and enabling characteristics, including senescent cells (3).

Telomeres, protective structures that “cap” the ends of linear chromosomes and preserve genome stability (4, 5), are major players in enabling replicative immortality. Due to the end-replication problem and a variety of lifestyle factors and stresses, telomere length erodes with cell division and aging, causing telomeres to shorten until reaching a critically shortened length, at which point they become dysfunctional and a permanent cell cycle arrest known as replicative senescence is entered (6–8).

Telomere shortening-induced senescence serves as an effective barrier to unlimited cell growth, and therefore represents an important tumor suppressor mechanism 9). However, senescence also underlies important phenotypes associated with aging and cancer; specifically, the senescence-associated secretory phenotype (SASP) promotes chronic inflammation and drives degenerative pathologies and carcinogenesis (10).

The cellular mortality enforced by progressive telomere shortening can be circumvented by telomerase, the specialized reverse transcriptase (TERT) whose integral RNA component (TERC) serves as a template for *de novo* addition of telomeric repeats onto newly replicated chromosomal termini (11, 12). However, telomerase activity is sufficient to maintain telomere length only in highly proliferative populations, such as germline and stem cells, and the vast majority of cancer cells in which mutations in the TERT promoter region or alternative splicing of the TERT transcript endow them with unlimited replicative potential (13, 14). Telomerase also has extracurricular activities associated with increasing cell proliferation, inhibiting anti-growth signaling, and activating invasion, therefore telomerase has also been proposed as a central regulator of all the hallmarks of cancer (15).

This special Research Topic highlights recent research and advances specifically related to the hallmark of replicative immortality. Articles in the collection cover a range of tumor types, including glioblastoma, pancreatic, gastric, colon, colorectal, and canine oral squamous cell carcinoma, and investigate key players such as telomerase activity, telomere length dynamics and regulation, and the immune landscape. The ultimate goal of all such studies is to identify actionable targets that inform targeted therapies aimed at countering tumor cell proliferation and immortality and improving treatment outcomes. Future studies will need to further investigate the emerging hallmarks and enabling characteristics, including the contributing and seemingly contradictory roles of senescent cells.

## Author contributions

The author confirms being the sole contributor of this work and has approved it for publication.
